# Dynamic of composite nanobeams resting on an elastic substrate with variable stiffness

**DOI:** 10.1016/j.heliyon.2024.e40168

**Published:** 2024-11-06

**Authors:** Dao Manh Lan, Pham Van Dong, M.A. Eltaher, Nguyen Trong Hai

**Affiliations:** a Hanoi University of Industry, No. 298 Cau Dien Street, Bac Tu Liem District, Hanoi City, Vietnam; b Mechanical Design and Production Department, Faculty of Engineering, Zagazig University, Ash Sharqiyah, Egypt; c Mechanical Engineering Department, Faculty of Engineering, King Abdulaziz University, Jeddah, Saudi Arabia; d HUTECH Institue of Engineering, HUTECH University, Ho Chi Minh City, Vietnam

**Keywords:** Dynamic, Composite, Nanobeams, FEM, Imperfection, Variable stiffness

## Abstract

This study introduces a novel approach by combining finite simulation and an enhanced shear deformation theory to analyze the dynamic behavior of multi-layer composite nanobeams supported by an elastic foundation. The calculation formulae are derived from nonlocal theory in order to account for the impact of size effect. An intriguing aspect of this research is the presence of intricate curved profiles in the two material layers of the beam. The elastic foundation exhibits varying stiffness throughout the beam's length, accounting for many imperfections in the beam and including the drag parameter of the material. These elements contribute to the steady evolution of the issue model towards more realistic structures. Nevertheless, the computation gets intricate and impedes the precise approach. However, our work has successfully resolved this difficult and significant difficulty using the two-node element. This research demonstrates the temporal evolution of the dynamic reactions of the point with the greatest displacement and velocity, as dictated by the law of change. Simultaneously, it also presents visual representations of every point on the beam as it undergoes temporal variations. These conclusions possess both scientific and practical value, establishing a foundation for real-world implementations.

## 1 Introduction

With the advancement of science and technology, the use of composite materials and structures constructed from such materials has seen a significant rise in their application in many aspects of life and technology. In order to optimize the functional capacities, scientists have developed composite constructions composed of many layers of materials. These structures provide a high stiffness-to-mass ratio and the capability to tailor material composition, providing designers with further freedom to apply them to particular tasks. This is also the reason that entices a significant number of scientists to investigate and compute the mechanical response of structures composed of composite materials. Asgharifard Sharabiani et al. [1] conducted a study on the non-linear free oscillations of functionally graded nanobeams, taking into account the influence of surface factors. Nazemnezhad and Hosseini-Hashemi [2] investigated the phenomenon of nonlocal nonlinear free vibration in functionally graded (FG) nanobeams. Li and coworkers [3] conducted research on the bending, buckling, and free vibration of a magneto electro elastic nanobeam using the principles of nonlocal theory. Tuna and Kirca [4] demonstrated the application of the finite element method to conduct bending, buckling, and free vibration analyses of Euler-Bernoulli nanobeams utilizing Eringen's nonlocal integral model. Emam et al. [5] investigated the phenomena of post-buckling and free vibration in nanobeams consisting of many layers, taking into account imperfections and a pre-existing stress load. Barretta and his team [6] investigated the free oscillations of elastic Timoshenko nano-beams using strain gradient and stress-driven nonlocal simulations. Borjalilou et al. [7] used an analytical approach to examine the flexing, collapsing, and independent oscillation of nonlocal FG-carbon nanotube-reinforced composite nanobeams. Arefi and coworkers [8,9] analyzed the oscillations of curved nanobeams made of FG polymer composite and strengthened with graphene nanoplatelets. The nanobeams were of different sizes and were supported by Pasternak foundations. Şimşek [10] presented closed-form results for the static, buckling, free, and forced oscillation of functionally graded nanobeams utilizing the nonlocal strain gradient concept. Nguyen Thai and his team [11] conducted a static bending study on symmetric three-layer FG sandwich beams. Each layer of the beams was composed of various functionally graded materials and they were joined together using shear connectors to allow for sliding motion. Tho et al. [12] introduced a finite element model that examines the flexoelectric impact on spinning nanobeams with geometric imperfections. Van Dung and coelleages [13] examined the free vibration response of micro FG beams while accounting for initial geometrical imperfection. Some related studies on static bending, buckling and specific vibration analysis of nano beams and plates can be referenced in these documents [[14], [15], [16], [17], [18], [19],[20], [21], [22], [23],[24], [25], [26], [27], [28], [29], [30], [31], [32], [33], [34], [35], [36],[37], [38], [39], [40], [41], [42], [43], [44], [45], [46], [47], [48], [49]].

When implementing beam structures, the stress exerted on composite beams may vary over time, resulting in intricate dynamic reactions of the beams that are influenced by several elements. In 2013, Uymaz [50] presented a nonlocal elasticity-based forced vibration analysis of functionally graded beams. Sahmani et al. [51] investigated how the surface properties affect the response of third-order shear deformable nanobeams under forced vibrations. Mareishi and coworkers [52] studied the response of smart two-phase nano-composite beams to external harmonic excitations, specifically focusing on nonlinear forced vibrations. Ansari et al. [53,54] conducted a study on the analysis of forced vibrations in Timoshenko nanobeams that are affected by size and nonlinearity. The study focused on the magneto-electro-thermo-elastic properties of the nanobeams and used the nonlocal elasticity theory. In addition, Ansari et al. [55] conducted a study on the analysis of forced vibrations in Timoshenko beams made of functionally graded carbon nanotube-reinforced composites, focusing on non-linear behavior. Akbas [56] analyzed the forced vibration behavior of nanobeams made of functionally graded materials using the modified couple stress theory, which takes into account the damping effect. These publications provide relevant research on the study of forced vibration in nano composite beams that may be referred to Refs. [[57], [58], [59], [60], [61], [62]].

Current investigations have shown that determining the mechanical response of multi-layer systems is of significant scientific importance. Nevertheless, these research are limited to conventional multi-layer structures, namely layers that are interconnected by faces that are parallel to the top and bottom faces of the structure. However, the examination of multi-layer systems with smooth or curved interfaces has not been conducted. The rationale for this is that the intersection of the material layers along the curve may enhance the structure's resistance. Therefore, it is important to thoroughly examine this matter. Consequently, this work presents a novel approach using the finite element technique to analyze the dynamic behavior of sandwich nanobeams. These nanobeams consist of interconnected material layers with sinusoidal and meandering curves, and the analysis also considers initial conformational defects. Consequently, doing analytical computations on these structures is very challenging and intricate.

The study is organized into the following primary areas. Section 2 provides a comprehensive explanation of the balancing equation and solution technique. Section 3 presents illustrative instances to validate the dependability of computational theory. Section 4 displays many instances of the dynamic response shown by composite beams. Section 5 outlines the significant findings of this study.

## 2 Balanced equation and solution method

**Fig. 1:**
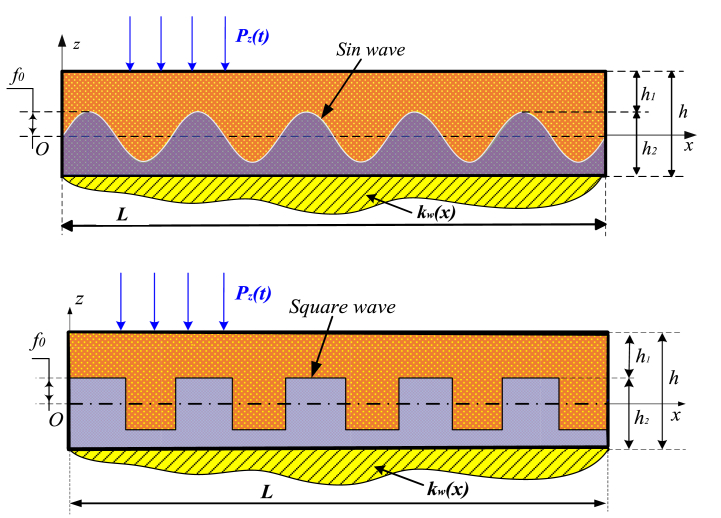
Composite nanobeam model subjected to time-dependent loading.

This study examines a composite nanobeam using the model shown in Fig. 1. The bean, which is supported by elastic foundations, exhibits variable stiffness characteristics based on its *x* coordinate. Additionally, it has geometric parameters such as length (*L*) and cross-sectional height (*h*). The beam consists of two layers of material with an uneven interface, and the height of each layer is denoted as *h*_*i*_ (*i* = 1–2).

The amplitude of the separation profile between the layers is *f*_0_ and the number of wavelengths is *n*, then the formula for the height *h*_*x*_ in both situations may be stated as follows. And this study presupposes the absence of separation between the material layers, hence making the issue more tractable since the displacement and deformation fields become continuous throughout the layers.

In case the boundary between two material layers has a sine waveform, one has:

(1) $$h_{x}=f_{0}\;\sin\left(2\frac{nx}{L}\pi \right)$$

and the case where the separation profile has a square wave waveform:

(2) $$h_{x}=\left\{ \begin{array}{l} f_{0}\;with\;\sin\left(2\frac{nx}{L}\pi \right)\geq 0\\ -f_{0}\;with\;\sin\left(2\frac{nx}{L}\pi \right)\leq 0 \end{array}\right.$$

Each layer of a composite beam is made from a porous material and the volume fraction of this porosity is given by the expression:

(3) $$\alpha_{m}^{\left(i\right)}=V_{0}\left\{1-k_{0}+\frac{\pi k_{0}}{2}\cos\left(\frac{\pi z}{h_{i}}\right)\right\}$$

where $\alpha_{m}^{\left(i\right)}$ represents the volume ratio of porosities in the *i*-th layer. The constant *k*_0_ represents the level of porosities in the material

At this time, the elastic modulus and density of the *i*-th layer depend on the pore volume ratio as follows:

(4) $$\begin{array}{l} E^{\left(i\right)}\left(z\right)=\left(1-\alpha_{m}^{\left(i\right)}\right)E_{0}^{\left(i\right)}\\ \rho^{\left(i\right)}\left(z\right)=\left(1-\alpha_{m}^{\left(i\right)}\right)\rho_{0}^{\left(i\right)} \end{array}$$

where $\rho_{0}^{\left(i\right)}$ represents the mass density and $E_{0}^{\left(i\right)}$ represents the elastic modulus in the *i*-th layer. This approach presupposes that the Poisson coefficient is uniform across all classes $\nu^{\left(1\right)}=\nu^{\left(2\right)}$.

The neutral axis and mean axis of the beam do not align due to the varying materials in the layers. The formula is used to determine the distance between these two axes.

(5) $$z_{d}=\frac{\int_{-h_{2}}^{h_{x}}E^{\left(2\right)}\left(z\right)zdz+\int_{h_{x}}^{h_{1}}E^{\left(1\right)}\left(z\right)zdz}{\int_{-h_{2}}^{h_{x}}E^{\left(2\right)}\left(z\right)dz+\int_{h_{x}}^{h_{1}}E^{\left(1\right)}\left(z\right)dz}$$

In order to provide computation expressions for dynamic issues, this study employs an enhanced shear deformation theory. The displacement field of the beam may be described by the following equation.

(6) $$\left\{ \begin{array}{l} d_{x}\left(x,y,z\right)=-\left(z-z_{d}\right)\frac{\partial d_{zb}}{\partial x}-r_{z}\frac{\partial d_{zs}}{\partial x}\\ d_{z}\left(x,y,z\right)=d_{zb}+d_{zs} \end{array}\right.$$

where *d*_*x*_ is the long displacement along the *x*-axis and *d*_*z*_ is the long displacement along the *z*-axis, and the function *r*_*z*_ has the form:

(7) $$r_{z}=\left(-\frac{4}{3}+\frac{5{\left(z-z_{d}\right)}^{3}}{3h^{2}}\right)$$

The formula is used to determine the longitudinal strain and shear strain of the beam, taking into account the imperfection of its original shape.

(8) $$\left\{ \begin{array}{l} \varsigma_{x}=\left(z-z_{d}\right)\varsigma_{z}+r_{z}\varsigma_{r}+\varsigma_{imp}\\ \varsigma_{xz}=s_{z}\varsigma_{s} \end{array}\right.$$

with the components specified as follows:

(9) $$\begin{array}{l} \varsigma_{z}=-\frac{\partial^{2}d_{zb}}{\partial x^{2}};\;\varsigma_{r}=-\frac{\partial^{2}d_{zs}}{\partial x^{2}};\varsigma_{imp}=\frac{\partial d_{zimp}}{\partial x}\frac{\partial \left(d_{zb}+d_{zs}\right)}{\partial x}\\ \varsigma_{s}=\frac{\partial d_{zs}}{\partial x};\;s_{z}=1-\frac{\partial r_{z}}{\partial z} \end{array}$$

where *d*_*zimp*_ is a parameter representing the imperfection of the shape. This study considers various types of imperfections through the following function [63].

(10) $$d_{zim}=\beta_{im}h\;\mathrm{sech}\left[\delta_{1}\left(\frac{x}{a}-\psi_{1}\right)\right]\cos\left[\mu_{1}\left(\frac{x}{a}-\psi_{1}\right)\right]$$

**Fig. 2:**
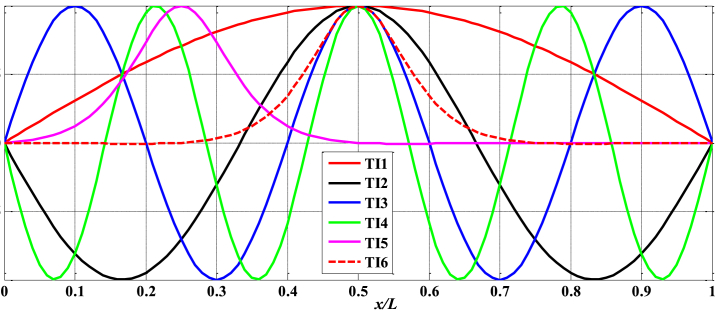
Imperfect forms of composite nanobeams.

Given that $\beta_{im}$ and $\delta_{1},\psi_{1}\text{,}$ and $\mu_{1}$ are constants denoting the degree of imperfection of the beam, changing these parameters will result in distinct deformations of the beam. This impacts the computational formulas of the beam, resulting in increased complexity of the calculations. However, this also enhances the fidelity of the issue simulation to the actual structure, hence increasing the significance of this work. This work gives six imperfect forms as shown in Fig. 2: Type 1 (TI1): $\left\{\delta_{1},\psi_{1},\mu \right\}$ = {0,1,0.5}, Type 2 (TI2): $\left\{\delta_{1},\psi_{1},\mu \right\}$ = {0,3,0.5}, Type 3 (TI3): $\left\{\delta_{1},\psi_{1},\mu \right\}$ = {0,5,0.5}, Type 4 (TI4): $\left\{\delta_{1},\psi_{1},\mu \right\}$ = {0,7,0.5}, Type 5 (TI5): $\left\{\delta_{1},\psi_{1},\mu \right\}$ = {15,2,0.25}, Type 6 (TI6): $\left\{\delta_{1},\psi_{1},\mu \right\}$ = {15,2,0.5}.

In this investigation, the size effect is taken into account in accordance with nonlocal theory, and the stress field in the *i*-th stratum of the beam is shaped by the consideration of shape defects [[64], [65], [66]].

(11) $$\begin{array}{l} \sigma_{x}^{\left(i\right)}-\lambda^{2}\frac{\partial^{2}\sigma_{x}^{\left(i\right)}}{\partial x^{2}}=E^{\left(i\right)}\left(\left(z-z_{d}\right)\varsigma_{z}^{\left(i\right)}+r_{z}\varsigma_{r}^{\left(i\right)}+\varsigma_{imp}^{\left(i\right)}\right)+\psi_{v}\frac{\partial }{\partial t}\left(\left(z-z_{d}\right)\varsigma_{z}^{\left(i\right)}+r_{z}\varsigma_{r}^{\left(i\right)}\right)\\ \tau_{xz}^{\left(i\right)}-\lambda^{2}\frac{\partial^{2}\tau_{xz}^{\left(i\right)}}{\partial x^{2}}=G^{\left(i\right)}\varsigma_{xz}^{\left(i\right)}+\psi_{v}\frac{\partial }{\partial t}\varsigma_{xz}^{\left(i\right)} \end{array}$$

where the nonlocal parameter is denoted by λ, which is also a characteristic of the internal drag parameter of the beam, $\psi_{v}$ is another parameter that demonstrates the impact of the size effect. In this context, $\sigma_{x}^{\left(i\right)}$ and $\tau_{xz}^{\left(i\right)}$ represents the normal stress and tangential stress of layer *i*, while $\nu^{\left(i\right)}$ and $E^{\left(i\right)}$ denote Poisson's coefficient and Young's modulus of elasticity of layer *i*, respectively, and $G^{\left(i\right)}=E^{\left(i\right)}/2\left(1+\nu^{\left(i\right)}\right)$.

Substituting Equation (9) into Equation (11), we obtain:

(12) $$\begin{array}{l} \left(1-l^{2}\nabla_{x}^{2}\right)\sigma_{x}^{\left(i\right)}=-\left(z-z_{d}\right)E^{\left(i\right)}\frac{\partial^{2}d_{zb}}{\partial x^{2}}-r_{z}E^{\left(i\right)}\frac{\partial^{2}d_{zs}}{\partial x^{2}}+E^{\left(i\right)}\frac{\partial d_{zimp}}{\partial x}\frac{\partial \left(d_{zb}+d_{zs}\right)}{\partial x}-\psi_{v}\frac{\partial }{\partial t}\left(\left(z-z_{d}\right)\frac{\partial^{2}d_{zb}}{\partial x^{2}}+r_{z}\frac{\partial^{2}d_{zs}}{\partial x^{2}}\right)\\ \left(1-l^{2}\nabla_{x}^{2}\right)\tau_{xz}^{\left(i\right)}=G^{\left(i\right)}s_{z}\frac{\partial d_{zs}}{\partial x}+\psi_{v}s_{z}\frac{\partial }{\partial t}\left(\frac{\partial d_{zs}}{\partial x}\right) \end{array}$$

After integrating over the thickness of the beam, the expressions for moment *M*_*x*_, higher moment *S*_*x*_ and shear force *Q*_*xz*_ are as follows:

(13) $$\begin{array}{c} M_{x}=\int_{-h_{2}}^{h_{x}}\left\{E^{\left(2\right)}{\left\{\varsigma_{x}\right\}}^{\left(2\right)}+\psi_{v}\frac{\partial }{\partial t}{\left\{\varsigma_{x}\right\}}^{\left(2\right)}\right\}\left(z-z_{d}\right)dz+\int_{h_{x}}^{h_{1}}\left\{E^{\left(1\right)}{\left\{\varsigma_{x}\right\}}^{\left(1\right)}+\psi_{v}\frac{\partial }{\partial t}{\left\{\varsigma_{x}\right\}}^{\left(1\right)}\right\}\left(z-z_{d}\right)dz\\ S_{x}=\int_{-h_{2}}^{h_{x}}\left\{E^{\left(2\right)}{\left\{\varsigma_{x}\right\}}^{\left(2\right)}+\psi_{v}\frac{\partial }{\partial t}{\left\{\varsigma_{x}\right\}}^{\left(2\right)}\right\}r_{z}dz+\int_{h_{x}}^{h_{1}}\left\{E^{\left(1\right)}{\left\{\varsigma_{x}\right\}}^{\left(1\right)}+\psi_{v}\frac{\partial }{\partial t}{\left\{\varsigma_{x}\right\}}^{\left(1\right)}\right\}r_{z}dz\\ Q_{xz}=\int_{-h_{2}}^{h_{x}}\left\{G^{\left(2\right)}{\left\{\varsigma_{xz}\right\}}^{\left(2\right)}+\psi_{v}\frac{\partial }{\partial t}{\left\{\varsigma_{xz}\right\}}^{\left(2\right)}\right\}dz+\int_{h_{x}}^{h_{1}}\left\{G^{\left(1\right)}{\left\{\varsigma_{xz}\right\}}^{\left(1\right)}+\psi_{v}\frac{\partial }{\partial t}{\left\{\varsigma_{xz}\right\}}^{\left(1\right)}\right\}dz \end{array}$$

Based on the nonlocal theory, one obtains the internal force components:

(14) $$\begin{array}{c} M_{x}-l^{2}\nabla_{x}^{2}M_{x}=\left\{\begin{array}{l} \int_{-h_{2}}^{h_{x}}E^{\left(2\right)}\left\{-\left(z-z_{d}\right)\frac{\partial^{2}d_{zb}}{\partial x^{2}}-r_{z}\frac{\partial^{2}d_{zs}}{\partial x^{2}}+\frac{\partial d_{zimp}}{\partial x}\frac{\partial \left(d_{zb}+d_{zs}\right)}{\partial x}\right\}\left(z-z_{d}\right)dz\\ +\int_{h_{x}}^{h_{1}}E^{\left(1\right)}\left\{-\left(z-z_{d}\right)\frac{\partial^{2}d_{zb}}{\partial x^{2}}-r_{z}\frac{\partial^{2}d_{zs}}{\partial x^{2}}+\frac{\partial d_{zimp}}{\partial x}\frac{\partial \left(d_{zb}+d_{zs}\right)}{\partial x}\right\}\left(z-z_{d}\right)dz\\ -\psi_{v}\int_{-h_{2}}^{h_{x}}\frac{\partial }{\partial t}\left(\left(z-z_{d}\right)\frac{\partial^{2}d_{zb}}{\partial x^{2}}+r_{z}\frac{\partial^{2}d_{zs}}{\partial x^{2}}\right)\left(z-z_{d}\right)dz\\ -\psi_{v}\int_{h_{x}}^{h_{1}}\frac{\partial }{\partial t}\left(\left(z-z_{d}\right)\frac{\partial^{2}d_{zb}}{\partial x^{2}}+r_{z}\frac{\partial^{2}d_{zs}}{\partial x^{2}}\right)\left(z-z_{d}\right)dz \end{array}\right\}=\left(D_{z}+\psi_{v}D_{zv}\frac{\partial }{\partial t}\right)\frac{\partial^{2}d_{zb}}{\partial x^{2}}+\left(D_{r}+\psi_{v}D_{rv}\frac{\partial }{\partial t}\right)\frac{\partial^{2}d_{zs}}{\partial x^{2}}+D_{imp}\frac{\partial d_{zimp}}{\partial x}\frac{\partial \left(d_{zb}+d_{zs}\right)}{\partial x}\\ S_{x}-l^{2}\nabla_{x}^{2}S_{x}=\left\{\begin{array}{l} \int_{-h_{2}}^{h_{x}}E^{\left(2\right)}\left\{-\left(z-z_{d}\right)\frac{\partial^{2}d_{zb}}{\partial x^{2}}-r_{z}\frac{\partial^{2}d_{zs}}{\partial x^{2}}+\frac{\partial d_{zimp}}{\partial x}\frac{\partial \left(d_{zb}+d_{zs}\right)}{\partial x}\right\}r_{z}dz\\ +\int_{h_{x}}^{h_{1}}E^{\left(1\right)}\left\{-\left(z-z_{d}\right)\frac{\partial^{2}d_{zb}}{\partial x^{2}}-r_{z}\frac{\partial^{2}d_{zs}}{\partial x^{2}}+\frac{\partial d_{zimp}}{\partial x}\frac{\partial \left(d_{zb}+d_{zs}\right)}{\partial x}\right\}r_{z}dz\\ -\psi_{v}\int_{-h_{2}}^{h_{x}}\frac{\partial }{\partial t}\left(\left(z-z_{d}\right)\frac{\partial^{2}d_{zb}}{\partial x^{2}}+r_{z}\frac{\partial^{2}d_{zs}}{\partial x^{2}}\right)r_{z}dz\\ -\psi_{v}\int_{h_{x}}^{h_{1}}\frac{\partial }{\partial t}\left(\left(z-z_{d}\right)\frac{\partial^{2}d_{zb}}{\partial x^{2}}+r_{z}\frac{\partial^{2}d_{zs}}{\partial x^{2}}\right)r_{z}dz \end{array}\right\}=\left(D_{r}+\psi_{v}D_{rv}\frac{\partial }{\partial t}\right)\frac{\partial^{2}d_{zb}}{\partial x^{2}}+\left(D_{rr}+\psi_{v}D_{rrv}\frac{\partial }{\partial t}\right)\frac{\partial^{2}d_{zs}}{\partial x^{2}}+D_{rimp}\frac{\partial d_{zimp}}{\partial x}\frac{\partial \left(d_{zb}+d_{zs}\right)}{\partial x}\\ Q_{xz}-l^{2}\nabla_{x}^{2}Q_{xz}=\left\{\int_{-h_{2}}^{h_{x}}G^{\left(2\right)}s_{z}^{2}dz+\int_{h_{x}}^{h_{1}}G^{\left(1\right)}s_{z}^{2}dz\right\}\left(\frac{\partial d_{zs}}{\partial x}\right)+\psi_{v}\left\{\int_{-h_{2}}^{h_{x}}s_{z}^{2}dz+\int_{h_{x}}^{h_{1}}s_{z}^{2}dz\right\}\frac{\partial }{\partial t}\left(\frac{\partial d_{zs}}{\partial x}\right)=\left(D_{s}+\psi_{v}D_{sv}\frac{\partial }{\partial t}\right)\frac{\partial d_{zs}}{\partial x} \end{array}$$

where the coefficients are calculated as follows:

(15) $$\begin{array}{c} D_{z}=-\left\{\int_{-h_{2}}^{h_{x}}E^{\left(2\right)}{\left(z-z_{d}\right)}^{2}dz+\int_{h_{x}}^{h_{1}}E^{\left(1\right)}{\left(z-z_{d}\right)}^{2}dz\right\}\text{;}\\ \begin{array}{c} D_{zv}=-\left\{\int_{-h_{2}}^{h_{x}}{\left(z-z_{d}\right)}^{2}dz+\int_{h_{x}}^{h_{1}}{\left(z-z_{d}\right)}^{2}dz\right\}\\ D_{r}=-\left\{\int_{-h_{2}}^{h_{x}}E^{\left(2\right)}r_{z}\left(z-z_{d}\right)dz+\int_{h_{x}}^{h_{1}}E^{\left(1\right)}r_{z}\left(z-z_{d}\right)dz\right\}\\ D_{rv}=-\left\{\int_{-h_{2}}^{h_{x}}r_{z}\left(z-z_{d}\right)dz+\int_{h_{x}}^{h_{1}}r_{z}\left(z-z_{d}\right)dz\right\} \end{array}\\ \begin{array}{c} D_{rr}=\left\{\int_{-h_{2}}^{h_{x}}E^{\left(2\right)}r_{z}^{2}dz+\int_{h_{x}}^{h_{1}}E^{\left(1\right)}r_{z}^{2}dz\right\};\;D_{rrv}=\left\{\int_{-h_{2}}^{h_{x}}r_{z}^{2}dz+\int_{h_{x}}^{h_{1}}r_{z}^{2}dz\right\}\\ D_{imp}=\left\{\int_{-h_{2}}^{h_{x}}E^{\left(2\right)}\left(z-z_{d}\right)dz+\int_{h_{x}}^{h_{1}}E^{\left(1\right)}\left(z-z_{d}\right)dz\right\}\\ \begin{array}{c} D_{rimp}=\left\{\int_{-h_{2}}^{h_{x}}E^{\left(2\right)}r_{z}dz+\int_{h_{x}}^{h_{1}}E^{\left(1\right)}r_{z}dz\right\};\;D_{s}=\left\{\int_{-h_{2}}^{h_{x}}G^{\left(2\right)}s_{z}^{2}dz+\int_{h_{x}}^{h_{1}}G^{\left(1\right)}s_{z}^{2}dz\right\}\\ D_{sv}=\left\{\int_{-h_{2}}^{h_{x}}s_{z}^{2}dz+\int_{h_{x}}^{h_{1}}s_{z}^{2}dz\right\} \end{array} \end{array} \end{array}$$

The Hamilton's principle is used to find the equilibrium equation of the nanobeam:

(16) $$\int_{t_{0}}^{t_{1}}\left(\delta \Pi_{load}-\delta \Pi_{beam}+\delta \Pi_{inertia}\right)dt=0$$

in which the components are specifically calculated as follows:

(17) $$\begin{array}{c} \delta \Pi_{beam}=\int_{\Omega }\int_{-h_{2}}^{h_{x}}\left(\sigma_{x}^{\left(2\right)}\delta \left(\varsigma_{x}^{\left(2\right)}\right)+\tau_{xz}^{\left(2\right)}\delta \left(\varsigma_{xz}^{\left(2\right)}\right)\right)dzd\Omega +\int_{\Omega }\int_{h_{x}}^{h_{1}}\left(\sigma_{x}^{\left(1\right)}\delta \left(\varsigma_{x}^{\left(1\right)}\right)+\tau_{xz}^{\left(1\right)}\delta \left(\varsigma_{xz}^{\left(1\right)}\right)\right)dzd\Omega +\int_{\Omega }\left\{k_{w}\left(1-t_{w}\;\sin\left(\frac{x}{L}\right)\right)d_{z}\delta \left(d_{z}\right)\right\}d\Omega \\ \begin{array}{l} =\int_{\Omega }\left(-M_{x}\frac{\partial^{2}\delta d_{zb}}{\partial x^{2}}-S_{x}\frac{\partial^{2}\delta d_{zs}}{\partial x^{2}}+Q_{xz}\frac{\partial \delta d_{zs}}{\partial x}\right)d\Omega \\ +\int_{\Omega }\left\{k_{w}\left(1-t_{w}\;\sin\left(\frac{x}{L}\right)\right)d_{z}\right\}\delta \left(d_{z}\right)d\Omega \end{array} \end{array}$$

where *k*_*w*_ and *t*_*w*_ are two elastic foundation parameters. This also implies that the foundation's rigidity varies in accordance with the length of the beams.

If *P*_*z*_(*t*) represents the external force acting on the beam, then its work can be expressed as:

(18) $$\delta \Pi_{f}=\int_{\Omega }P_{z}\left(t\right)\delta d_{z}dA=\int_{\Omega }P_{z}\left(t\right)\delta \left(d_{zb}+d_{zs}\right)d\Omega$$

The work done by the force of inertia is as follows:

(19) $$\delta \Pi_{inertia}=\int_{\Omega }\int_{-h_{2}}^{h_{x}}\rho^{\left(2\right)}\left(z\right)\left(\frac{\partial^{2}d_{x}}{\partial t^{2}}\delta d_{x}+\frac{\partial^{2}d_{z}}{\partial t^{2}}\delta d_{z}\right)dzd\Omega +\int_{\Omega }\int_{h_{x}}^{h_{1}}\rho^{\left(1\right)}\left(z\right)\left(\frac{\partial^{2}d_{x}}{\partial t^{2}}\delta d_{x}+\frac{\partial^{2}d_{z}}{\partial t^{2}}\delta d_{z}\right)dzd\Omega =\int_{\Omega }\left\{\begin{array}{l} R_{0}\left(\frac{\partial^{2}d_{zb}}{\partial t^{2}}+\frac{\partial^{2}d_{zs}}{\partial t^{2}}\right)\delta \left(d_{zb}+d_{zs}\right)+R_{1}\frac{\partial^{2}d_{zb,xx}}{\partial t^{2}}\delta d_{zb}+R_{2}\frac{\partial^{2}d_{zs,xx}}{\partial t^{2}}\delta d_{zb}\\ +R_{2}\frac{\partial^{2}d_{zb,xx}}{\partial t^{2}}\delta d_{zs}+R_{3}\frac{\partial^{2}d_{zs,xx}}{\partial t^{2}}\delta d_{zs} \end{array}\right\}d\Omega$$

in which

(20) $$\left\{R_{0},R_{1},R_{2},R_{3}\right\}=\int_{-h_{2}}^{h_{x}}\rho^{\left(2\right)}\left(z\right)\left\{1,{\left(z-z_{d}\right)}^{2},\left(z-z_{d}\right)r_{z},r_{z}^{2}\right\}dz+\int_{h_{x}}^{h_{1}}\rho^{\left(1\right)}\left(z\right)\left\{1,{\left(z-z_{d}\right)}^{2},\left(z-z_{d}\right)r_{z},r_{z}^{2}\right\}dz$$

Combining Equations (16), (17), (18), (19), the equilibrium equation of the beam in two variables has the following form:

(21) $$\begin{array}{c} \delta d_{zb}:\frac{\partial^{2}M_{x}}{\partial x^{2}}+R_{0}\left(\frac{\partial^{2}d_{zb}}{\partial t^{2}}+\frac{\partial^{2}d_{zs}}{\partial t^{2}}\right)+R_{1}\frac{\partial^{2}d_{zb,xx}}{\partial t^{2}}+R_{2}\frac{\partial^{2}d_{zs,xx}}{\partial t^{2}}+k_{w}\left(1-t_{w}\;\sin\left(\frac{x}{L}\right)\right)d_{z}+P_{z}\left(t\right)=0\\ \delta d_{zs}:\frac{\partial^{2}S_{x}}{\partial x^{2}}+\frac{\partial Q_{xz}}{\partial x}+R_{0}\left(\frac{\partial^{2}d_{zb}}{\partial t^{2}}+\frac{\partial^{2}d_{zs}}{\partial t^{2}}\right)+R_{2}\frac{\partial^{2}d_{zb,xx}}{\partial t^{2}}+R_{3}\frac{\partial^{2}d_{zs,xx}}{\partial t^{2}}+k_{w}\left(1-t_{w}\;\sin\left(\frac{x}{L}\right)\right)d_{z}+P_{z}\left(t\right)=0 \end{array}$$

With attention to Equation (14), Equation (21) is rewritten as follows:

(22) $$\begin{array}{c} \frac{\partial^{2}M_{x}}{\partial x^{2}}+\left(1-l^{2}\nabla_{x}^{2}\right)P_{z}\left(t\right)+\left(1-l^{2}\nabla_{x}^{2}\right)\left\{R_{0}\left(\frac{\partial^{2}d_{zb}}{\partial t^{2}}+\frac{\partial^{2}d_{zs}}{\partial t^{2}}\right)+R_{1}\nabla_{x}^{2}\frac{\partial^{2}d_{zb}}{\partial t^{2}}+R_{2}\nabla_{x}^{2}\frac{\partial^{2}d_{zs}}{\partial t^{2}}\right\}+\left(1-l^{2}\nabla_{x}^{2}\right)\left\{k_{w}\left(1-t_{kw}\;\sin\left(\frac{x}{L}\right)\right)d_{z}\right\}=0\\ \frac{\partial^{2}S_{x}}{\partial x^{2}}+\frac{\partial Q_{xz}}{\partial x}+\left(1-l^{2}\nabla_{x}^{2}\right)P_{z}\left(t\right)+\left(1-l^{2}\nabla_{x}^{2}\right)\left\{\begin{array}{l} R_{0}\left(\frac{\partial^{2}d_{zb}}{\partial t^{2}}+\frac{\partial^{2}d_{zs}}{\partial t^{2}}\right)\\ +R_{2}\nabla_{x}^{2}\frac{\partial^{2}d_{zb}}{\partial t^{2}}+R_{3}\nabla_{x}^{2}\frac{\partial^{2}d_{zs}}{\partial t^{2}} \end{array}\right\}+\left(1-l^{2}\nabla_{x}^{2}\right)\left\{k_{w}\left(1-t_{kw}\;\sin\left(\frac{x}{L}\right)\right)d_{z}\right\}=0 \end{array}$$

Examining Equation (22), it is evident that it is a complex equation with coefficients that are influenced by the *x* coordinate of the elastic foundation and the imperfect form. Additionally, these coefficients vary based on the changing law of the thickness of each layer. Consequently, this poses a significant difficulty for analytical approaches. Thus, this study employs the finite simulation approach.

Both sides of equation (22) are multiplied by the corresponding variations, one obtains:

(23) $$\begin{array}{c} \int_{\Omega }\left(\begin{array}{l} \frac{\partial^{2}M_{x}}{\partial x^{2}}+\left(1-l^{2}\nabla_{x}^{2}\right)P_{z}\left(t\right)+\left(1-l^{2}\nabla_{x}^{2}\right)\left\{\begin{array}{l} R_{0}\left(\frac{\partial^{2}d_{zb}}{\partial t^{2}}+\frac{\partial^{2}d_{zs}}{\partial t^{2}}\right)+R_{1}\nabla_{x}^{2}\frac{\partial^{2}d_{zb}}{\partial t^{2}}\\ +R_{2}\nabla_{x}^{2}\frac{\partial^{2}d_{zs}}{\partial t^{2}} \end{array}\right\}\\ +\left(1-l^{2}\nabla_{x}^{2}\right)\left\{k_{w}\left(1-t_{kw}\;\sin\left(\frac{x}{L}\right)\right)d_{z}\right\} \end{array}\right)\delta d_{zb}d\Omega =0\\ \int_{\Omega }\left(\begin{array}{l} \frac{\partial^{2}S_{x}}{\partial x^{2}}+\frac{\partial Q_{xz}}{\partial x}+\left(1-l^{2}\nabla_{x}^{2}\right)P_{z}\left(t\right)+\left(1-l^{2}\nabla_{x}^{2}\right)\left\{\begin{array}{l} R_{0}\left(\frac{\partial^{2}d_{zb}}{\partial t^{2}}+\frac{\partial^{2}d_{zs}}{\partial t^{2}}\right)\\ +R_{2}\nabla_{x}^{2}\frac{\partial^{2}d_{zb}}{\partial t^{2}}+R_{3}\nabla_{x}^{2}\frac{\partial^{2}d_{zs}}{\partial t^{2}} \end{array}\right\}\\ +\left(1-l^{2}\nabla_{x}^{2}\right)\left\{k_{w}\left(1-t_{kw}\;\sin\left(\frac{x}{L}\right)\right)d_{z}\right\} \end{array}\right)\delta d_{zs}d\Omega =0 \end{array}$$

The beam is subdivided into two-node elements, each node having four degrees of freedom:

(24) $$d_{e}=\sum_{i=1}^{2}{\left\{d_{zbi},d_{zsi},{\left(\frac{\partial d_{zb}}{\partial x}\right)}_{i},{\left(\frac{\partial d_{zs}}{\partial x}\right)}_{i}\right\}}^{T}$$

This work conducts interpolation as follows:

(25) $$\begin{array}{c} \left[d_{zb},d_{zs}\right]=\sum_{i=1}^{2}\left\{{ϒ}_{i}\left[d_{zbi},d_{zsi}\right]+{ϒ}_{i+1}\left[{\left(\frac{\partial d_{zb}}{\partial x}\right)}_{i},{\left(\frac{\partial d_{zs}}{\partial x}\right)}_{i}\right]\right\}=\left[\Psi_{b},\Psi_{s}\right]d_{e}\\ \left[\frac{\partial d_{zb}}{\partial x},\frac{\partial d_{zs}}{\partial x}\right]=\sum_{i=1}^{2}\left\{\frac{\partial {ϒ}_{i}}{\partial x}\left[d_{zbi},d_{zsi}\right]+\frac{\partial {ϒ}_{i+1}}{\partial x}\left[{\left(\frac{\partial d_{zb}}{\partial x}\right)}_{i},{\left(\frac{\partial d_{zs}}{\partial x}\right)}_{i}\right]\right\}=\left[\Psi_{bx},\Psi_{sx}\right]d_{e}\\ \left[\frac{\partial^{2}d_{zb}}{\partial x^{2}},\frac{\partial^{2}d_{zs}}{\partial x^{2}}\right]=\sum_{i=1}^{2}\left\{\frac{\partial^{2}{ϒ}_{i}}{\partial x^{2}}\left[d_{zbi},d_{zsi}\right]+\frac{\partial^{2}{ϒ}_{i+1}}{\partial x^{2}}\left[{\left(\frac{\partial d_{zb}}{\partial x}\right)}_{i},{\left(\frac{\partial d_{zs}}{\partial x}\right)}_{i}\right]\right\}=\left[\Psi_{b2x},\Psi_{s2x}\right]d_{e} \end{array}$$

where ${ϒ}_{i}$ are the corresponding Hercmit functions, and displacement vector at a point belonging to the interpolated element:

(26) $$d={\left\{d_{zb},d_{zs},\left(\frac{\partial d_{zb}}{\partial x}\right),\left(\frac{\partial d_{zs}}{\partial x}\right)\right\}}^{T}=\left\{\Psi_{b},\Psi_{s},\Psi_{bx},\Psi_{sx}\right\}d_{e}=\Psi d_{e}$$

Eq. (9) is:

(27) $$\begin{array}{c} \varsigma_{z}=-\frac{\partial^{2}d_{zb}}{\partial x^{2}}=-\Psi_{b2x}d_{e};\;\varsigma_{r}=-\frac{\partial^{2}d_{zs}}{\partial x^{2}}=-\Psi_{s2x}d_{e}\text{;}\\ \varsigma_{imp}=\frac{\partial d_{zimp}}{\partial x}\left(\Psi_{bx}+\Psi_{sx}\right)d_{e}=\Psi_{imp}d_{e}\\ \varsigma_{s}=\frac{\partial d_{zs}}{\partial x}=\Psi_{sx}d_{e} \end{array}$$

Substituting Equation (27) into Equation (23), the dynamic equation of the beam is written in finite simulation form as follows:

(28) $$\sum_{e=1}^{m}M_{e}\frac{\partial^{2}d_{3e}\left(t\right)}{\partial t^{2}}+\sum_{e=1}^{m}C_{e}\frac{\partial d_{3e}\left(t\right)}{\partial t}+\sum_{e=1}^{m}K_{e}d_{3e}\left(t\right)=\sum_{e=1}^{m}F_{e}\left(t\right)$$

where *m* is the number of elements, ***M***_***e***_ is the element mass matrix, ***C***_***e***_ is the element resistance matrix, ***K***_***e***_ is the element stiffness matrix, ***F***_***e***_ is the element load vector, their expressions are as follows:

(29) $$\begin{array}{c} K_{e}=\int_{\Omega_{e}}\left\{\left(\begin{array}{l} \Psi_{b2x}^{T}D_{z}\Psi_{b2x}+\Psi_{b2x}^{T}D_{r}\Psi_{s2x}+\Psi_{s2x}^{T}D_{r}\Psi_{b2x}+\Psi_{s2x}^{T}D_{rr}\Psi_{s2x}\\ +\Psi_{b2x}^{T}D_{imp}\Psi_{imp}+\Psi_{imp}^{T}D_{imp}\Psi_{b2x}+\Psi_{s2x}^{T}D_{rimp}\Psi_{imp}+\Psi_{imp}^{T}D_{rimp}\Psi_{s2x}\\ +\Psi_{imp}^{T}{\bar{D}}_{imp}\Psi_{imp}+\Psi_{sx}^{T}D_{s}\Psi_{sx}\\ +\left(1-l^{2}\nabla_{x}^{2}\right)k_{w}\left(1-t_{kw}\;\sin\left(\frac{x}{L}\right)\right){\left(\Psi_{b}+\Psi_{s}\right)}^{T}\left(\Psi_{b}+\Psi_{s}\right) \end{array}\right)\right\}d\Omega \\ C_{e}=\psi_{v}\int_{\Omega_{e}}\left(\begin{array}{l} \Psi_{2bx}^{T}D_{zv}\Psi_{2bx}+\Psi_{2bx}^{T}D_{rv}\Psi_{2sx}+\Psi_{2sx}^{T}D_{rv}\Psi_{2bx}+\Psi_{2sx}^{T}D_{rrv}\Psi_{2sx}\\ \Psi_{sx}^{T}D_{sv}\Psi_{sx}+\Psi_{imp}^{T}D_{zimpv}\Psi_{2bx}+\Psi_{imp}^{T}D_{rimpv}\Psi_{2sx} \end{array}\right)d\Omega \\ \begin{array}{c} M_{e}=\int_{\Omega_{e}}\int_{-h_{2}}^{h_{x}}\left(1-l^{2}\nabla_{x}^{2}\right)\rho^{\left(2\right)}\left(z\right)\left({Χ}^{T}\Psi^{T}\Psi Χ\right)dzdV+\int_{\Omega }\int_{h_{x}}^{h_{1}}\left(1-l^{2}\nabla_{x}^{2}\right)\rho^{\left(1\right)}\left(z\right)\left({Χ}^{T}\Psi^{T}\Psi Χ\right)dzd\Omega \\ F_{e}=\int_{\Omega_{e}}\left(1-l^{2}\nabla_{x}^{2}\right)p\left(\Psi_{b}+\Psi_{s}\right)d\Omega \end{array} \end{array}$$

in which

(30) $$\begin{array}{c} D_{zimpv}=\left\{\int_{-h_{2}}^{h_{x}}\left(z-z_{d}\right)dz+\int_{h_{x}}^{h_{1}}\left(z-z_{d}\right)dz\right\}\\ D_{rimpv}=\left\{\int_{-h_{2}}^{h_{x}}r_{z}dz+\int_{h_{x}}^{h_{1}}r_{z}dz\right\}\text{;}\\ Χ=\left[\begin{array}{cccc} 0 & 0 & -\left(z-z_{d}\right) & -\left(-\frac{4}{3}+\frac{5{\left(z-z_{d}\right)}^{3}}{3h^{2}}\right)\\ 1 & 1 & 0 & 0 \end{array}\right] \end{array}$$

The computation of the element stiffness matrix, as per formula (29), is executed using the Gauss quadrature technique with two integration points, eliminating the need for the shear correction factor and the reduced integral.

The undamped natural vibration equation of the nanobeam may be expressed in the following manner, assuming no applied external force and neglecting the drag coefficient:

(31) $$\left\{\sum_{e=1}^{m}K_{e}-\omega^{2}\sum_{e=1}^{m}M_{e}\right\}\sum_{e}d_{e}=0$$

By disregarding the components that include the time factor in Equation (28), the static equilibrium equation of the composite beam may be simplified to the following form:

(32) $$\sum_{e}K_{e}d_{e}=\sum_{e}F_{e}$$

If one end of the beam has a single support boundary condition, then this work is denoted S, where two boundary conditions need to be constrained: $\left\{d_{zbi}=0,d_{zsi}=0\right\}$. If one end of the beam is subject to a clamp connection, denoted C, four boundary conditions need to be constrained: $\left\{d_{zbi}=0,d_{zsi}=0,{\left(\frac{\partial d_{zb}}{\partial x}\right)}_{i}=0,{\left(\frac{\partial d_{zs}}{\partial x}\right)}_{i}=0\right\}$. If the beam end is designated as "free" (F), no boundary requirements need to be imposed. The beam may be represented as S-S, indicating two single ends. C-S represents one fixed end and one single supporting end. C-C denotes a double-ended beam. C-F signifies that one end of the beam is clamped while the other end is free.

The dynamic responses of composite nanobeams are influenced by several elements such as the elastic foundation, contact profile features between material layers, drag parameters, imperfect forms, and boundary conditions. These factors determine the element matrices of the nanobeams. This work aims to provide intriguing research findings.

## 3 Verification examples

**Fig. 3:**
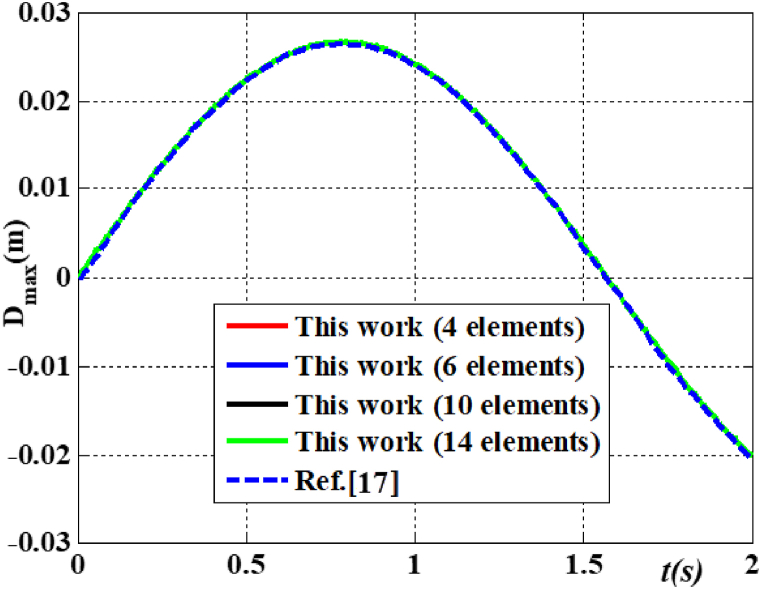
Displacement at free beam end of CF beam with different dividing meshes.

Example 1 The beam's dimensions are as follows: *L* = 1 m, h *=* 0.1 m, b *=* 0.1 m, E *=* 151 GPa, ρ = 3000 kg/m^3^, and ν = 0.2882 [17]. The beam is characterized by boundary conditions CF and a variable harmonic load at the free end (P = 10^3^sin(2*t*) kN). Fig. 3 illustrates the displacement of the free end under various grid configurations. The figure illustrates that the calculation outcomes of this study closely align with those presented in the document [17]. To facilitate the calculations, a lattice consisting of ten elements was chosen for this research.

Example 2 Consider a beam with length L = 10 nm, h = 0.1*L* and h *=* 0.05L, E *=* 30 GPa, ν = 0.3, and ρ = 1 kg/m^3^ [67,68]. The beam is subjected to a uniformly distributed load with intensity *q*_0_ = 1 N/m. The two parameters displacement and frequency used for comparison are:

(33) $$w_{\max}^{*}=100d_{zmax}\frac{EI}{q_{0}L^{4}};\;\omega^{*}=\omega_{1}L^{2}\sqrt{\frac{\rho A}{EI}}$$

Table 1, Table 2 display the computed findings and provide a comparison of the beam's displacement and natural vibration frequency. It is evident that our effort yields comparable computational outcomes to those reported in publications [67,68].

Example 3 Consider a nanobeam with length L = 10000 nm, L/h = 20, E = 390 GPa, ν = 0.3, and ρ = 3960 kg/m^3^ [69]. The ith natural oscillation frequency is defined:

(34) $$C_{fi}=\omega_{i}L^{2}\sqrt{\frac{\rho A}{EI}}$$

**Table 1:** Maximum displacement of nanobeams with different nonlocal parameters, S-S, CB: classic beam, TB: Timoshenko beam.

	$\lambda^{2}$ (nm^2^)	CB [67]	CB [68]	TB [67]	TB [68]	This work
*L* = 10*h*	0	1.313	1.302	1.348	1.334	1.334
	0.5	1.380	1.364	1.421	1.397	1.397
	1.0	1.448	1.427	1.493	1.459	1.461
	1.5	1.516	1.489	1.566	1.522	1.525
	2.0	1.584	1.552	1.639	1.584	1.588
*L* = 20*h*	0	1.313	1.302	1.321	1.310	1.309
	0.5	1.380	1.364	1.390	1.372	1.372
	1.0	1.448	1.427	1.460	1.435	1.435
	1.5	1.516	1.489	1.529	1.497	1.497
	2.0	1.584	1.552	1.598	1.560	1.560

**Table 2:** Frequency of nanobeams with different nonlocal parameters, S-S, CB: classic beam, TB: Timoshenko beam.

	$\lambda^{2}$ (nm^2^)	CB [68]	CB [68]	TB [68]	TB [68]	This work
*L* = 10*h*	0	9.869	9.869	9.745	9.705	9.708
	0.5	9.634	9.634	9.513	9.474	9.477
	1.0	9.415	9.415	9.297	9.259	9.261
	1.5	9.211	9.211	9.095	9.058	9.060
	2.0	9.019	9.019	8.905	8.869	8.871
*L* = 20*h*	0	9.869	9.869	9.838	9.848	9.828
	0.5	9.634	9.634	9.604	9.594	9.594
	1.0	9.415	9.415	9.385	9.376	9.376
	1.5	9.211	9.211	9.181	9.172	9.173
	2.0	9.019	9.019	8.990	8.981	8.982

Table 3 presents the calculation results and comparison with data published in the document [69] for the first five natural oscillation frequencies. This once again proves the reliability of this work.

**Table 3:** Frequency $C_{fi}$ of nanobeams with different values of λ.

$\lambda^{2}$ (10^−12^ m^2^)		$C_{f1}$	$C_{f2}$	$C_{f3}$	$C_{f4}$	$C_{f5}$
0	Present work	9.8286	38.8332	85.6778	148.4383	224.8913
	Eltaher et al. [69]	9.8797	39.6419	89.6599	160.5776	217.6917
1	Present work	9.3768	32.8832	62.3780	92.5872	121.3172
	Eltaher et al. [69]	9.4238	33.4875	64.6769	97.9683	131.0893
2	Present work	8.9821	29.0313	51.4486	72.9427	92.7875
	Eltaher et al. [69]	9.0257	29.5254	53.1705	76.7870	99.6006
3	Present work	8.6333	26.2776	44.7847	62.1074	78.0011
	Eltaher et al. [69]	8.6741	26.7022	46.2062	65.2317	83.5032

## 4 Numerical results

This part presents the calculation results of the dynamic response of the composite nanobeam, based on the calculation theory discussed in the previous section. The length of the beam, denoted as *L*, is fixed at 10000 nm throughout the calculation. The height of the cross-section is also considered *h* = *L*/10 and *L*/20. The mechanical properties of the upper and lower layers are [69,70] *E*_1_ = 70 GPa, $\nu_{1}=0.3$, $\rho_{1}$ = 2700 kg/m^3^, *E*_2_ = 390 GPa, $\nu_{2}=0.3$, $\rho_{2}$ = 3960 kg/m^3^, and *h*_1_ = 0.6*h*, *h*_2_ = 0.4*h*, *h*_0_ = *h*_1_/2, and the drag coefficient $\beta_{v}=\frac{\psi_{v}}{E_{1}}$.

The load is evenly distributed on the beam and changes over time according to the law:

(35) $$P_{z}\left(t\right)=\mu_{0}.q\left(t\right)$$

with amplitude $\mu_{0}$ = 0.5 N, and the function q(t) shows the time-varying law of the load, this work considers the following two cases:

Sinusoidal load:

(36) $$q\left(t\right)=\left\{ \begin{array}{l} \sin\left(\omega_{load}\frac{t}{t_{1}}\right)\;0\leq t\leq t_{1}\\ 0\;t>t_{1} \end{array}\right.$$

with *t*_1_ = 43 ns.

Triangular load:

(37) $$q\left(t\right)=\left\{ \begin{array}{l} 1-\frac{t}{t_{1}}\;0\leq t\leq t_{1}\\ 0\;t>t_{1} \end{array}\right.$$

The dynamic response of the beam shown in this study is displacement and velocity, which are normalized as follows:

(38) $$C_{d}=\frac{10E_{2}h_{0}^{3}}{\mu_{0}L^{4}}u_{z};\;C_{v}=\frac{10h_{0}^{3}}{\mu_{0}L^{3}}\sqrt{\rho_{2}E_{2}};\;h_{0}=L/10$$

**Fig. 4:**
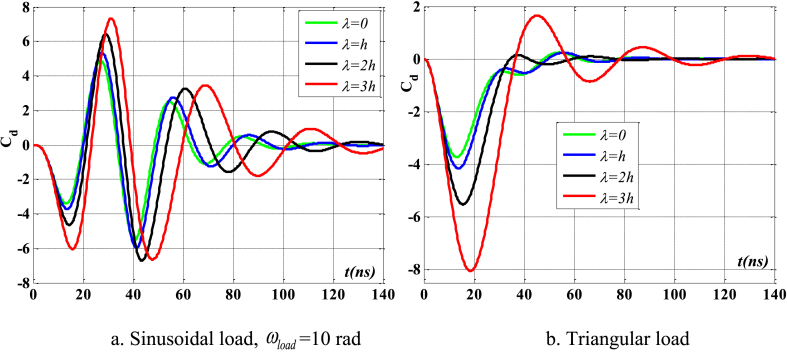
The maximum displacement of the beam varies with time and depends on λ, *h*_0_ = 0.5*h*_1_, *L*/*h* = 10, sin wave, *V*_0_ = 0.2, *k* = 0.2, *n* = 0.5, $t_{kw}$ = 0.5, TI1, S-S, $\beta_{v}$ = 5.10^−9^.

**Fig. 5:**
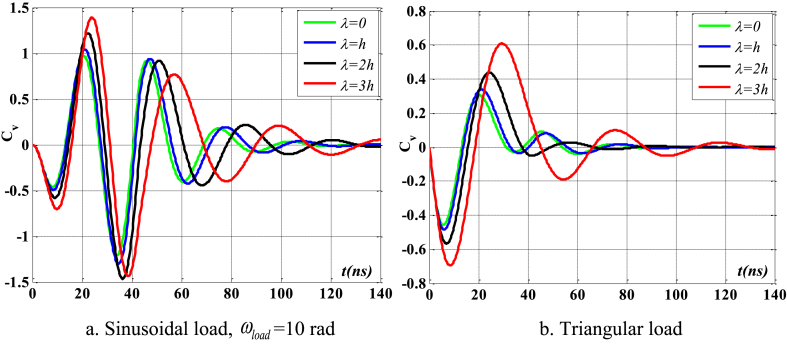
The maximum velocity of the beam varies with time and depends on λ, *h*_0_ = 0.5*h*_1_, *L*/*h* = 10, sin wave, *V*_0_ = 0.2, *k* = 0.2, *n* = 0.5, $t_{kw}$ = 0.5, TI1, S-S, $\beta_{v}$ = 5.10^−9^.

**Fig. 6:**
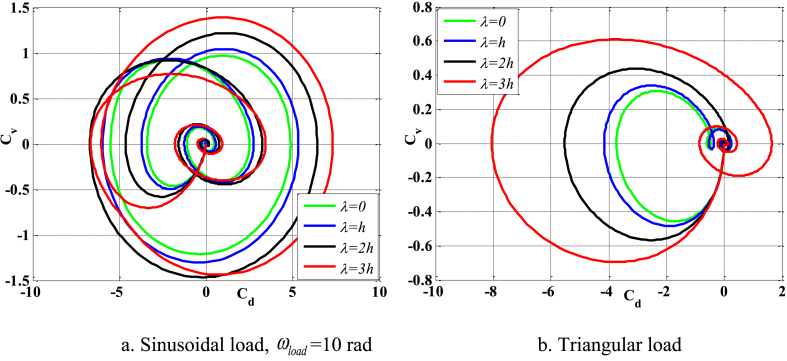
Phase diagram depends on λ, *h*_0_ = 0.5*h*_1_, *L*/*h* = 10, sin wave, *V*_0_ = 0.2, *k* = 0.2, *n* = 0.5, $t_{kw}$ = 0.5, TI1, S-S, $\beta_{v}$ = 5.10^−9^.

**Fig. 7:**
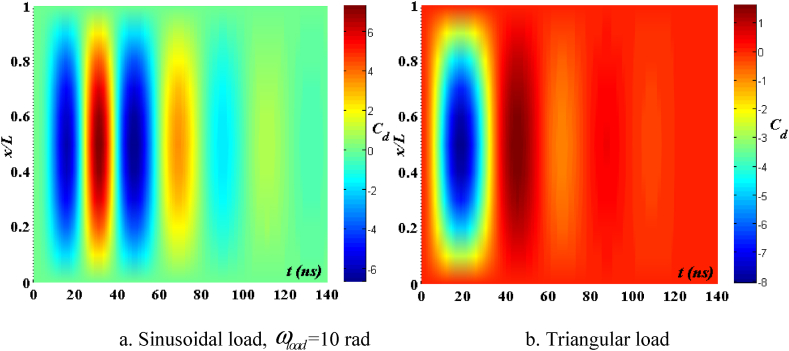
The displacement of the beam changes over time, λ = 3*h*, *L*/*h* = 10, *h*_0_ = 0.5*h*_1_, sin wave, *V*_0_ = 0.2, *k* = 0.2, *n* = 0.5, $t_{kw}$ = 0.5, TI1, S-S, $\beta_{v}$ = 5.10^−9^.

Fig. 4, Fig. 5 depict the temporal changes in maximum displacement and maximum velocity for several instances of the nonlocal parameter λ. The phase diagram in Fig. 6 represents several values of λ and two distinct load types. As the value of λ increases, both the maximum displacement and maximum velocity of the beam also rise. This occurs because an increase in the parameter's value results in a greater gap between the molecules of the beam material, hence reducing the beam's stiffness, which in turn causes an increase in displacement and velocity. Simultaneously, parameter λ also influences the duration of damping for both displacement and velocity. Fig. 6's phase diagram demonstrates that at the point of greatest displacement, the velocity is at a value of zero. Nevertheless, as the beam continues to oscillate, the structural drag coefficient induces a steady oscillation in the beam, resulting in the persistence of displacement even after the velocity reaches its maximum. The viscous damping parameter leads to the dissipation of the beam's vibrational energy, resulting in a progressive attenuation of the beam's vibrations. Fig. 7 depicts the temporal progression of every location on the beam. It is evident that varying loading patterns result in distinct displacement response shapes at each point on the beam.

**Fig. 8:**
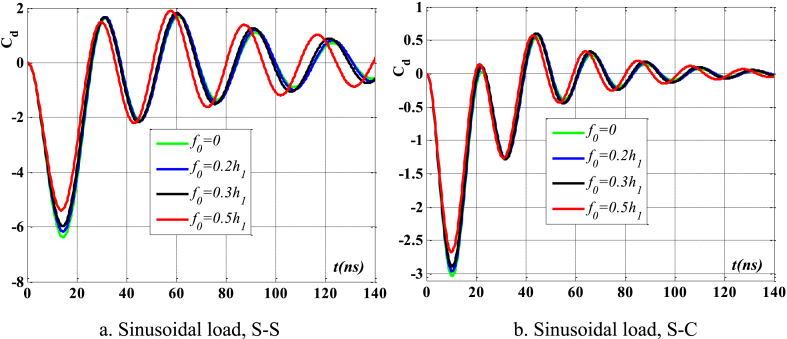
The maximum displacement of the beam varies with time and depends on *f*_0_, λ = *h*, *L*/*h* = 10, sin wave, *V*_0_ = 0.2, *k* = 0.2, *n* = 0.5, $t_{kw}$ = 0.5, TI1, $\beta_{v}$ = 1.10^−9^.

**Fig. 9:**
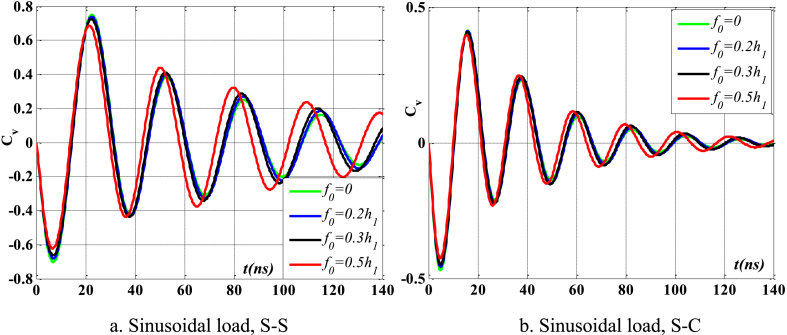
The maximum velocity of the beam varies with time and depends on *f*_0_, λ = *h*, *L*/*h* = 10, sin wave, *V*_0_ = 0.2, *k* = 0.2, *n* = 0.5, $t_{kw}$ = 0.5, TI1, $\beta_{v}$ = 1.10^−9^.

**Fig. 10:**
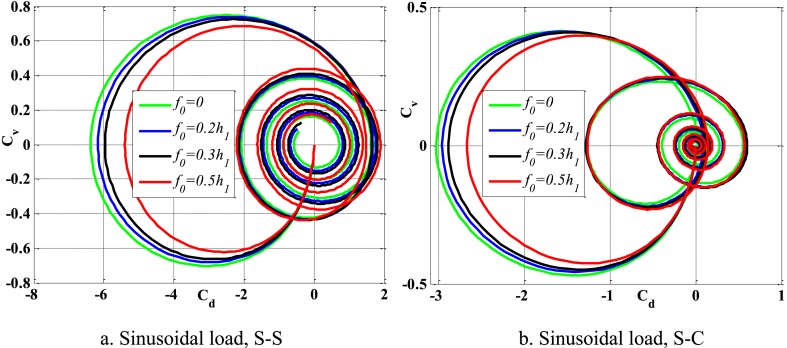
Phase diagram depends on *f*_0_, λ = *h*, *L*/*h* = 10, sin wave, *V*_0_ = 0.2, *k* = 0.2, *n* = 0.5, $t_{kw}$ = 0.5, TI1, $\beta_{v}$ = 1.10^−9^.

**Fig. 11:**
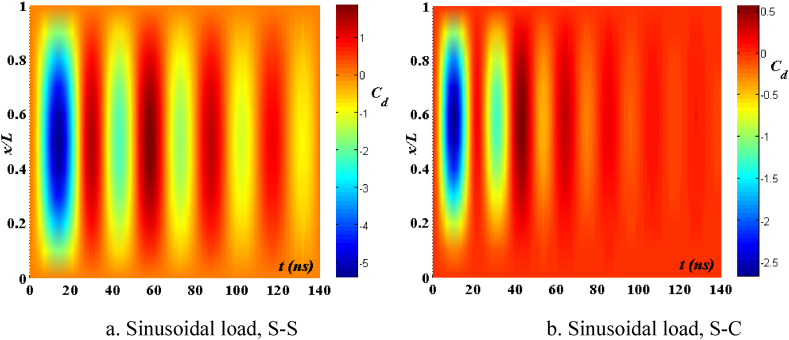
The displacement of the beam changes over time, *f*_0_ = 0.5*h*_1_, λ = *h*, *L*/*h* = 10, sin wave, *V*_0_ = 0.2, *k* = 0.2, *n* = 0.5, $t_{kw}$ = 0.5, TI1, $\beta_{v}$ = 1.10^−9^.

Fig. 8, Fig. 9 depict the relationship between amplitude *f*_0_ and the fluctuation of maximum displacement and maximum velocity over time. Fig. 10 displays the phase diagram for this particular scenario. Upon examining these findings, it is evident that as the amplitude *f*_0_ of the contact surface grows, there is a reduction in both the maximum displacement and maximum velocity of the beam. Furthermore, the level of attenuation of beam oscillations over time also varies. The displacement of points on the beam over time differs significantly between S-S beams and S-C beams. Fig. 11 provides a vivid demonstration of this.

**Fig. 12:**
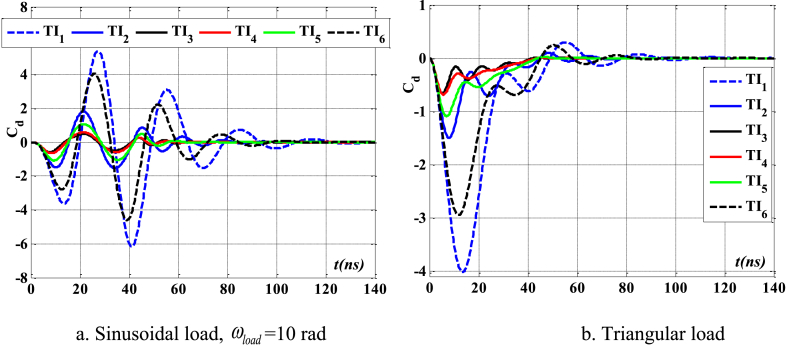
The maximum displacement of the beam depends on the imperfect, square wave form, λ = *h*, *h*_0_ = 0.5*h*_1_, *L*/*h* = 10, *V*_0_ = 0.2, *k* = 0.2, *n* = 0.5, $t_{kw}$ = 0.5, S-S, $\beta_{v}$ = 5.10^−9^.

**Fig. 13:**
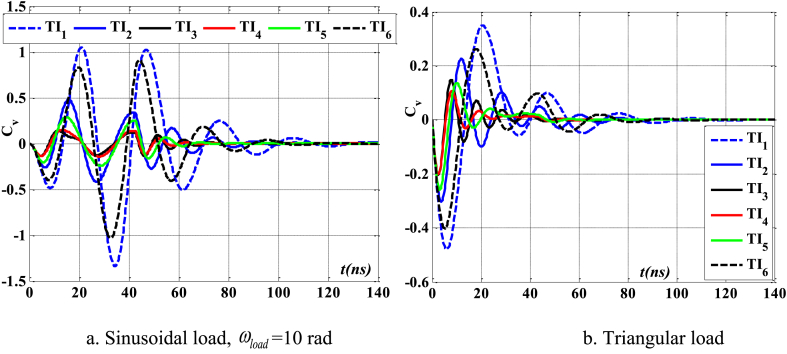
The maximum velocity of the beam depends on the imperfect, square wave form, λ = *h*, *h*_0_ = 0.5*h*_1_, *L*/*h* = 10, *V*_0_ = 0.2, *k* = 0.2, *n* = 0.5, $t_{kw}$ = 0.5, S-S, $\beta_{v}$ = 5.10^−9^.

**Fig. 14:**
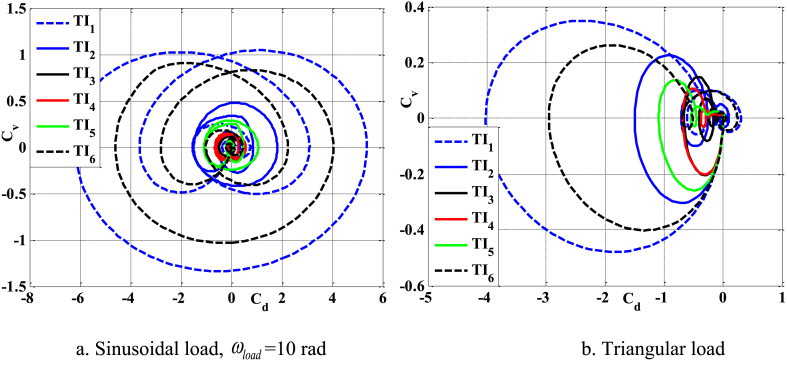
The phase diagram depends on the imperfect, square wave form, λ = *h*, *h*_0_ = 0.5*h*_1_, *L*/*h* = 10, *V*_0_ = 0.2, *k* = 0.2, *n* = 0.5, $t_{kw}$ = 0.5, S-S, $\beta_{v}$ = 5.10^−9^.

**Fig. 15:**
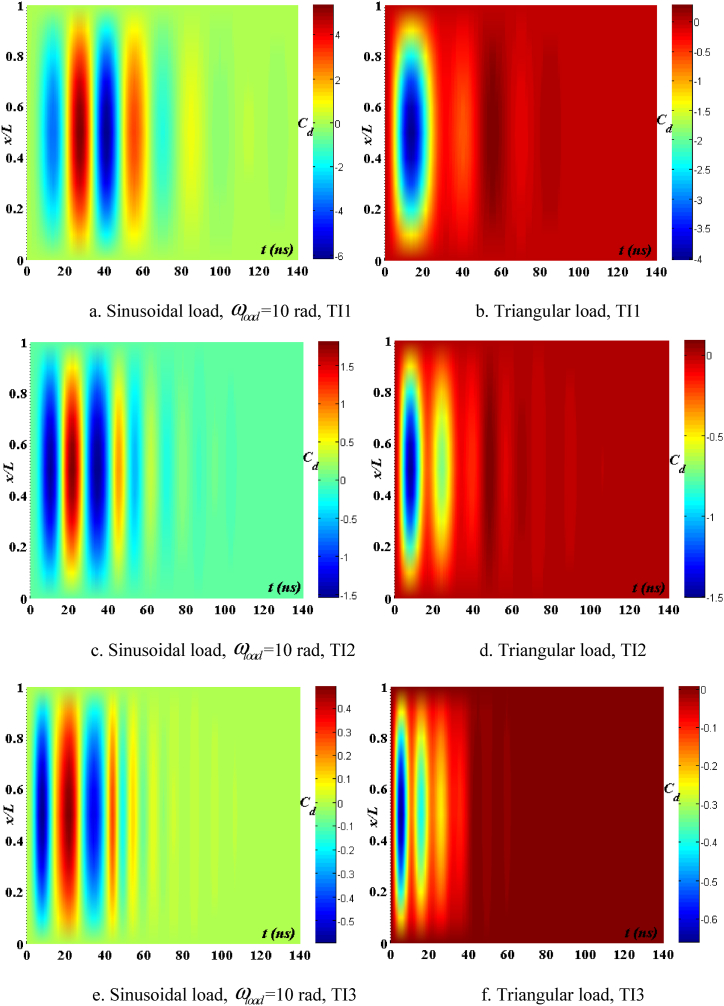

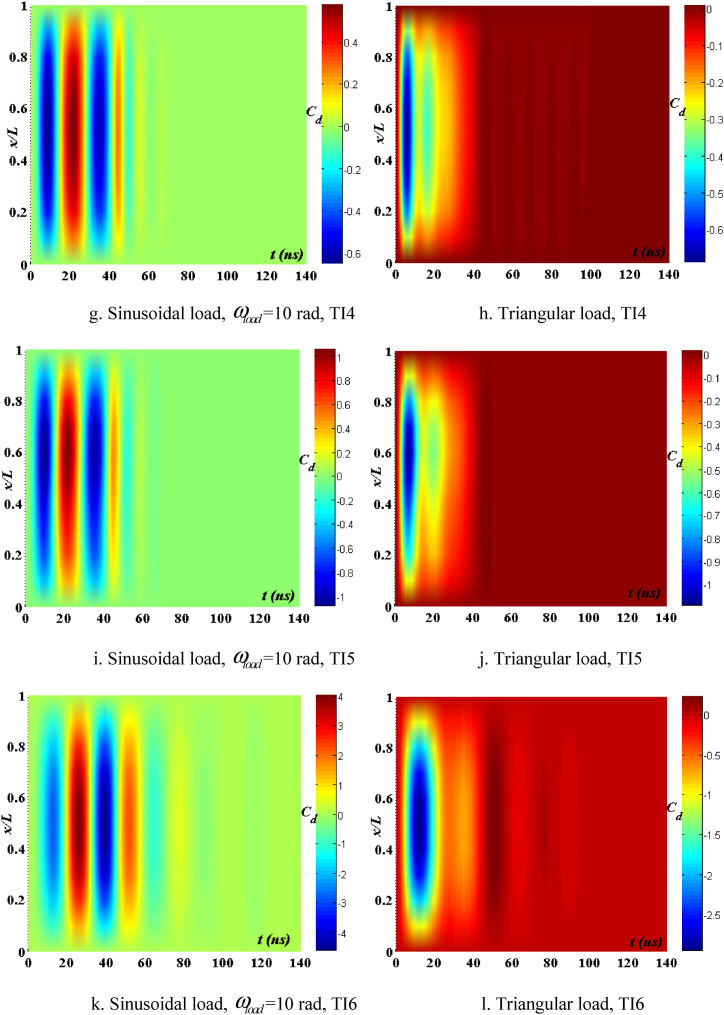
The beam's displacement changes over time, square wave, λ = *h*, *L*/*h* = 10, *h*_0_ = 0.5*h*_1_, *V*_0_ = 0.2, *k* = 0.2, *n* = 0.5, $t_{kw}$ = 0.5, S-S, $\beta_{v}$ = 5.10^−9^.

Fig. 12, Fig. 13, Fig. 14 demonstrate the impact of flawed shapes on the peak velocity and displacement response over time. When beams exhibit varying flawed forms, the magnitudes of displacement and velocity likewise fluctuate. The timing of the peak value of these reactions varies, as does the rate of attenuation with time. The reason for this is that the presence of the defective shape alters the rigidity of the beam, hence modifying its reaction. Fig. 15 depicts the temporal progression of beams exhibiting various forms of defects. The findings shown in this image provide more evidence that the distorted form significantly influences the temporal displacement response of the beam.

**Fig. 16:**
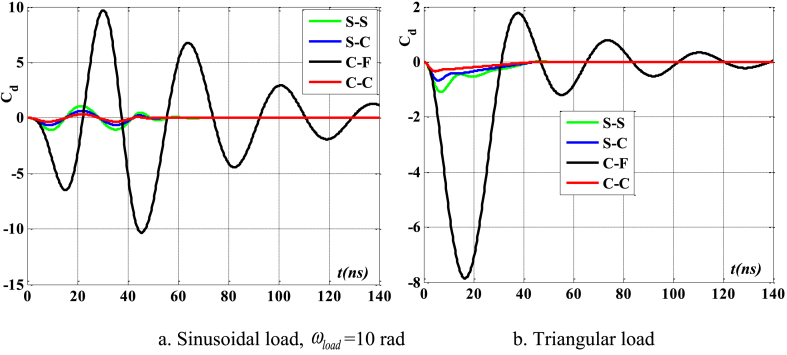
The maximum displacement of the beam depends on the boundary conditions, square wave, TI5, λ = *h*, *h*_0_ = 0.5*h*_1_, *L*/*h* = 10, *V*_0_ = 0.2, *k* = 0.2, *n* = 0.5, $t_{kw}$ = 0.5, S-S, $\beta_{v}$ = 5.10^−9^.

**Fig. 17:**
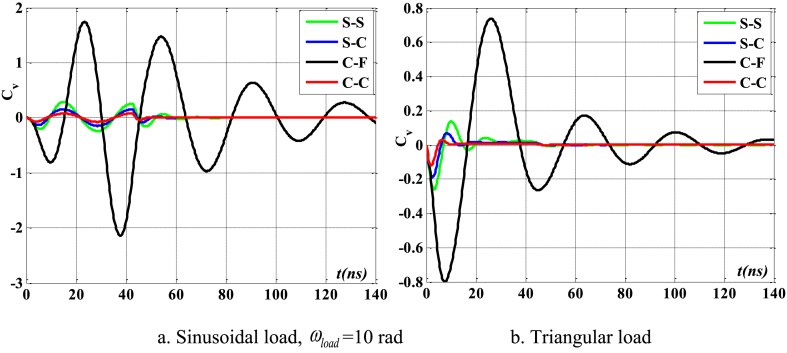
The maximum velocity of the beam depends on the boundary conditions, square wave, TI5, λ = *h*, *h*_0_ = 0.5*h*_1_, *L*/*h* = 10, *V*_0_ = 0.2, *k* = 0.2, *n* = 0.5, $t_{kw}$ = 0.5, S-S, $\beta_{v}$ = 5.10^−9^.

**Fig. 18:**
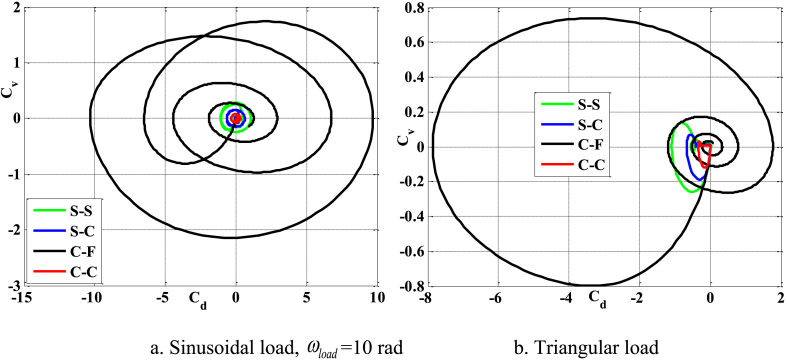
The phase diagram depends on the boundary conditions, TI5, square wave, λ = *h*, *h*_0_ = 0.5*h*_1_, *L*/*h* = 10, *V*_0_ = 0.2, *k* = 0.2, *n* = 0.5, $t_{kw}$ = 0.5, $\beta_{v}$ = 5.10^−9^.

**Fig. 19:**
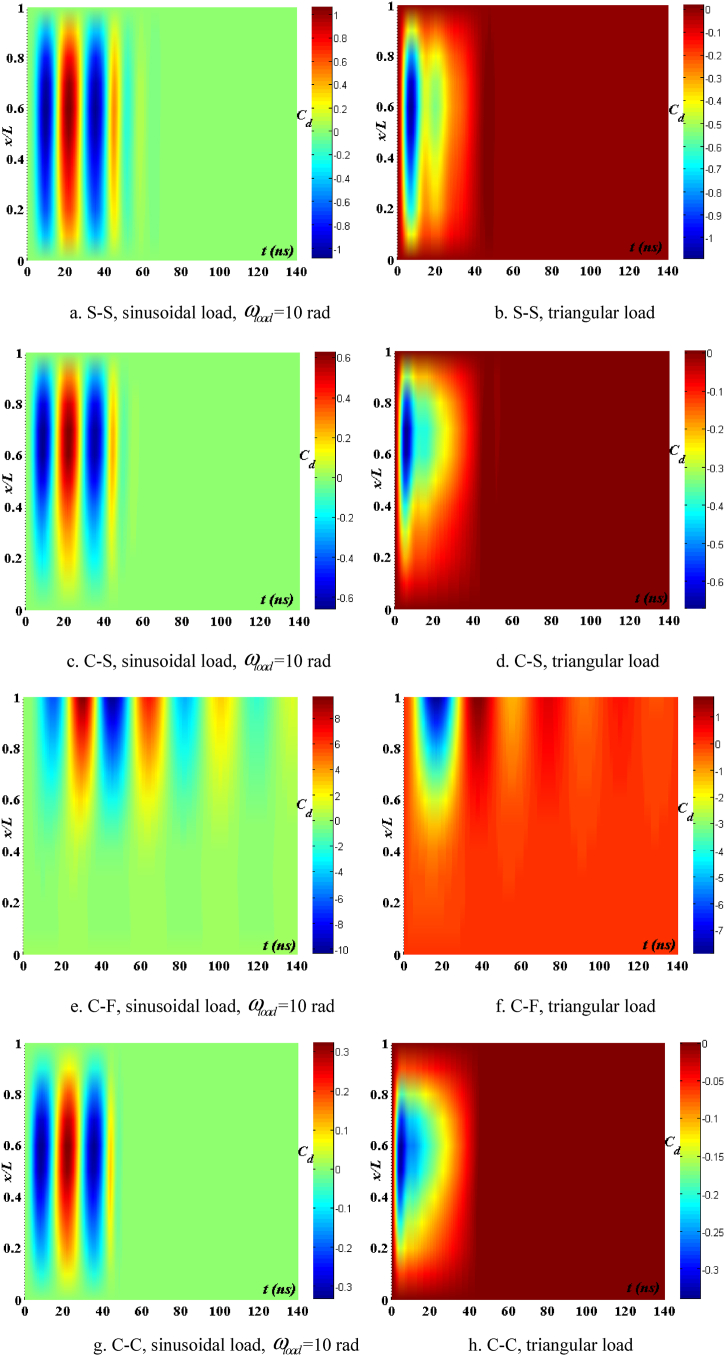
The displacement of the beam varies over time with different boundary conditions, square wave, TI5, λ = *h*, *L*/*h* = 10, *h*_0_ = 0.5*h*_1_, *V*_0_ = 0.2, *k* = 0.2, *n* = 0.5, $t_{kw}$ = 0.5, $\beta_{v}$ = 5.10^−9^.

Fig. 16, Fig. 17, Fig. 18 illustrate the boundary conditions that impact the displacement and velocity response of the beam. C-C beams exhibit the least displacement and velocity values, whereas C-F beams demonstrate the greatest displacement and velocity values. Furthermore, the period at which the maximum value of the velocity and displacement responses occurs is also influenced by boundary conditions. The degree of damping over time of such reactions is likewise influenced by boundary conditions. Fig. 19 clearly demonstrates the impact of boundary circumstances on the temporal displacement response of the beam.

**Fig. 20:**
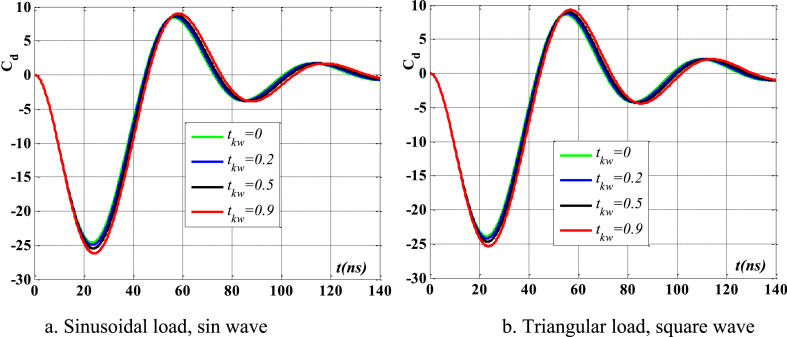
The maximum displacement of the beam varies with time depending on *t*_*kw*_, *f*_0_ = 0.5*h*_1_, λ = *h*, *L*/*h* = 20, *V*_0_ = 0.2, *k* = 0.2, *n* = 0.5, TI1, S-S, $\beta_{v}$ = 1.10^−8^.

**Fig. 21:**
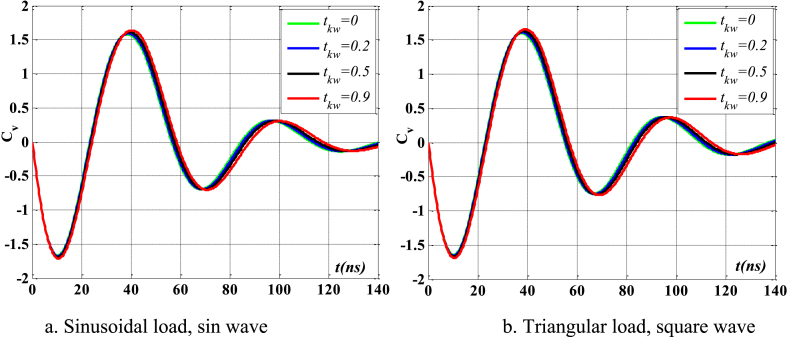
The maximum velocity of the beam changes over time depending on *t*_*kw*_, *f*_0_ = 0.5*h*_1_, λ = *h*, *L*/*h* = 20, *V*_0_ = 0.2, *k* = 0.2, *N* = 0.5, TI1, S-S, $\beta_{v}$ = 1.10^−8^.

**Fig. 22:**
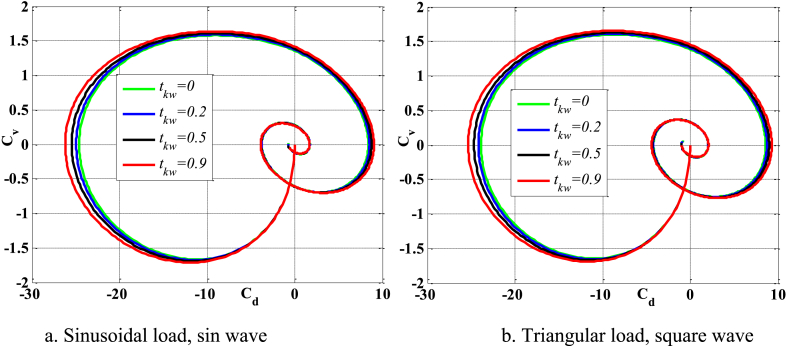
Phase diagram depends on *t*_*kw*_, *f*_0_ = 0.5*h*_1_, λ = *h*, *L*/*h* = 20, *V*_0_ = 0.2, *k* = 0.2, *n* = 0.5, TI1, S-S, $\beta_{v}$ = 1.10^−8^.

**Fig. 23:**
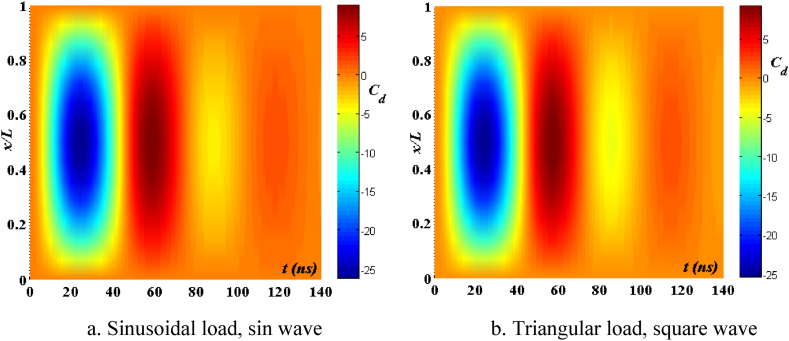
The displacement of the beam changes over time, *t*_*kw*_ = 0.9, *f*_0_ = 0.5*h*_1_, λ = *h*, *L*/*h* = 20, *V*_0_ = 0.2, *k* = 0.2, *n* = 0.5, TI1, S-S, $\beta_{v}$ = 1.10^−8^.

Fig. 20, Fig. 21, Fig. 22 depict the temporal behavior of the beam's displacement and velocity, which vary based on the characteristics of the elastic basis. As the value of $t_{kw}$ grows, the beam's maximum displacement and velocity both increase. The stiffness of the elastic basis reduces as the parameter $t_{kw}$ increases, however, the impact of $t_{kw}$ is quite insignificant. The displacement and velocity of the beam exhibit little variation whether subjected to a sinusoidal contact profile or a square wave contact profile. This is further shown in Fig. 23.

**Fig. 24:**
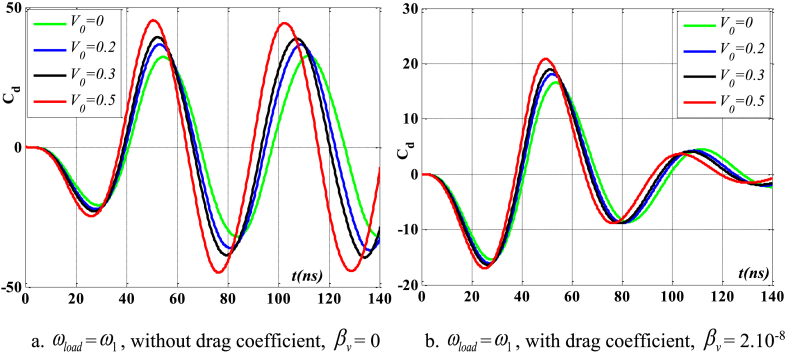
Maximum displacement varies with time and depends on *V*_*0*_, sinusoidal load, square wave, *t*_*kw*_ = 0.5, *f*_0_ = 0.5*h*_1_, λ = *h*, *L*/*h* = 10, *k* = 0.2, *n* = 0.5, TI1, C-F.

**Fig. 25:**
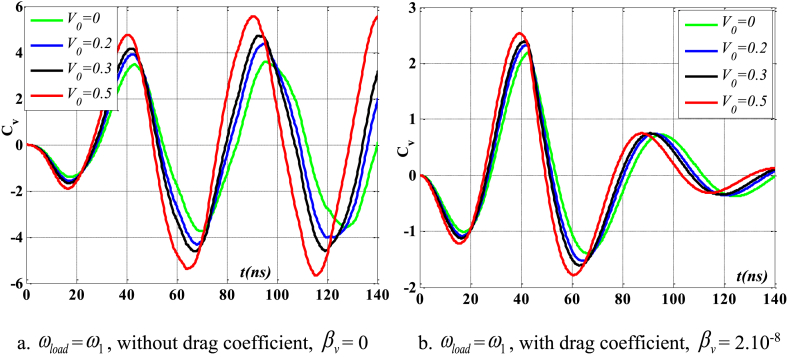
Maximum velocity varies with time and depends on *V*_*0*_, sinusoidal load, square wave, *t*_*kw*_ = 0.5, *f*_0_ = 0.5*h*_1_, λ = *h*, *L*/*h* = 10, *k* = 0.2, *n* = 0.5, TI1, C-F.

**Fig. 26:**
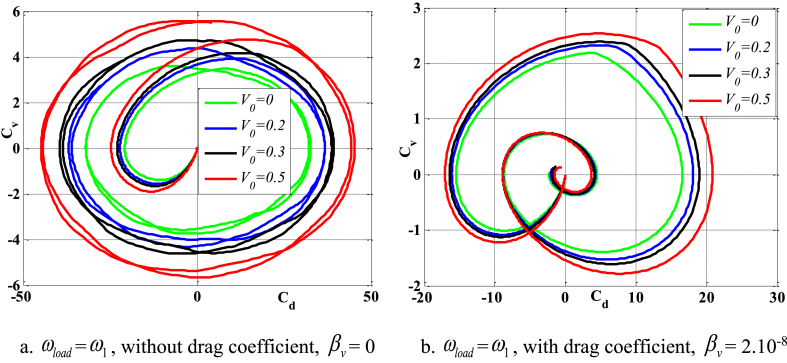
Phase diagram depends on *V*_*0*_, sinusoidal load, square wave, *t*_*kw*_ = 0.5, *f*_0_ = 0.5*h*_1_, λ = *h*, *L*/*h* = 10, *k* = 0.2, *n* = 0.5, TI1, C-F.

**Fig. 27:**
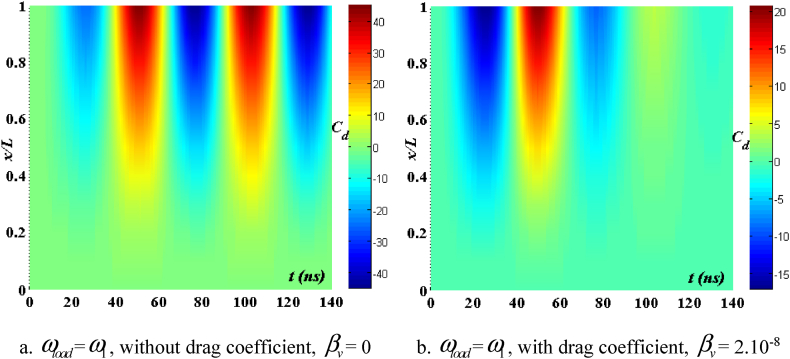
The displacement of the beam changes over time, sinusoidal load, square wave, *t*_*kw*_ = 0.5, *f*_0_ = 0.5*h*_1_, λ = *h*, *L*/*h* = 10, *V*_0_ = 0.5, *k* = 0.2, *n* = 0.5, TI1, C-F.

Fig. 24, Fig. 25, Fig. 26, Fig. 27 illustrate the beam's dynamic response under two conditions: with and without drag coefficient, where the frequency of the external pushing force matches the beam's natural vibration frequency. Comments are displayed in the following manner: In the absence of a drag coefficient after the application of an external force, the beam continues to vibrate with a significant amplitude and does not experience any damping. Upon cessation of the external force, the velocity and displacement exhibit a phase difference of 90^0^. However, when considering the drag coefficient, the velocity of the beam gradually decreases to zero as a response to displacement, once the external force ceases to act. Additionally, due to the impact of the drag coefficient, the velocity and displacement are only out of phase by 90^0^ at the point where displacement reaches its maximum value. Concurrently, when the parameter *V*_0_ grows, both displacement and velocity rise, resulting in a weakened beam due to the increased void ratio of the material.

## 5 Conclusion

This study investigates the dynamic behavior of multi-layer composite nanobeams supported by a variable stiffness elastic foundation, including the drag coefficient, using the recently enhanced shear deformation theory. This study is noteworthy and innovative as it demonstrates the connection of mechanical behavior, formulates the equilibrium equation, and resolves it using the finite element simulation approach. This research also discusses the presence of several sorts of initial form imperfections. The equilibrium equation was derived using the nonlocal theory, and the solution was obtained using a two-nodal element. The primary deductions are derived as follows.

- The drag coefficient of the structure results in the slow attenuation of the beam's vibration in the absence of any external force. The level of damping is primarily influenced by the material properties, boundary conditions, and the nature of the load exerted on the beam.
- The larger the value of the nonlocal parameter, the lower the stiffness of the beam, so the larger the displacement and velocity.
- The imperfect form and type of boundary conditions affect both the maximum value of displacement and velocity as well as the shape of the displacement and velocity responses over time.

The finite element technique may effectively address increasingly intricate issues, including nonlinear difficulties, fatigue phenomena, and the failure of nano-beam constructions. This research serves as the foundation for investigating further aspects of multi-layer composite nanostructures, including layer separation and material optimization, among others. Furthermore, they may be very advantageous when used in both daily life and technological contexts.

## CRediT authorship contribution statement

**Dao Manh Lan:** Writing – original draft, Validation, Methodology, Conceptualization. **Pham Van Dong:** Validation, Supervision, Project administration, Funding acquisition, Conceptualization. **M.A. Eltaher:** Writing – original draft, Project administration, Investigation, Conceptualization. **Nguyen Trong Hai:** Writing – review & editing, Supervision, Formal analysis.

## Data availability

The article contains the data used to support the results of the research.

## Declaration of competing interest

The authors declare the following financial interests/personal relationships which may be considered as potential competing interests:

There are no conflicts to declare.
